# Supplementary material for: Genome-wide analysis of the WRKY genes and their important roles during cold stress in white clover
Source: PeerJ. 2023 Jul 11;11:e15610. doi: 10.7717/peerj.15610 (PMC10348312; doi:10.7717/peerj.15610)
Supplement: Supplemental Information 1 [file peerj-11-15610-s001.docx]

**Table S1 Primers used for qRT-PCR analysis of the TrWRKY genes**

| **Name** | **Forward primer sequences (5’→3’)** | **Reverse primer sequences (5’→3’)** |
| --- | --- | --- |
| TrWRKY039 | AGGAAGAGAAAAGCTGAGAGT | GGAGCACCTAAAGTAAGCTC |
| TrWRKY041 | GCTATGCTATTGCTGAGAGC | CCTAGGAGAGGGGTTATCTC |
| TrWRKY079 | TATCCTGTGGATGATGCAGT | GATCCACCTCGCTTTTAGTC |
| TrWRKY084 | GGTGAAACCCTTCTTAACTCG | AGCAAATCTTGGTTCCTTTTG |
| TrWRKY100 | CTTTCGCATAGTCTAACGCT | TTAGACGGTCCAGACAAGAG |
| TrWRKY101 | CTTTCGCATAGTCTAACGCT | TTAGACGGTCCAGACAAGAG |
| TrWRKY113 | AACCTTGCTACCCTCTTTCA | AAGGTGAACCCTTTATCGGT |
| Action | TGCTTGATTCCGGTGATGGTGTG | TTCTCGGCAGAGGTACTGAAGGAG |
