# Supplementary material for: Genome-wide analysis of the WRKY genes and their important roles during cold stress in white clover
Source: PeerJ. 2023 Jul 11;11:e15610. doi: 10.7717/peerj.15610 (PMC10348312; doi:10.7717/peerj.15610)
Supplement: Supplemental Information 2 [file peerj-11-15610-s002.doc]

**Table S2 Ka/Ks values of *TrWRKY* gene pairs in the white clover**

| **Gene_1** | **Gene_1** | **Ka** | **Ks** | **Ka/Ks** |
| --- | --- | --- | --- | --- |
| TrWRKY001 | TrWRKY003 | 0.441665763 | 1.205485231 | 0.366380069 |
| TrWRKY003 | TrWRKY090 | 0.683267528 | 2.440210677 | 0.280003499 |
| TrWRKY006 | TrWRKY044 | 0.769285627 | 2.011891217 | 0.382369395 |
| TrWRKY006 | TrWRKY089 | 0.556620896 | 2.787077185 | 0.19971492 |
| TrWRKY008 | TrWRKY011 | 0.46210266 | 1.808379016 | 0.255534186 |
| TrWRKY010 | TrWRKY009 | 0.014836056 | 0.067842833 | 0.218682735 |
| TrWRKY010 | TrWRKY105 | 0.702191546 | 2.176066196 | 0.322688504 |
| TrWRKY011 | TrWRKY018 | 0.542254918 | 2.19436388 | 0.247112579 |
| TrWRKY012 | TrWRKY047 | 0.792302157 | 1.169720779 | 0.677342979 |
| TrWRKY014 | TrWRKY018 | 0.007102221 | 0.058386264 | 0.121641978 |
| TrWRKY015 | TrWRKY019 | 0.08963135 | 0.163644975 | 0.547718313 |
| TrWRKY017 | TrWRKY016 | 0.004202575 | 0.053216579 | 0.078971151 |
| TrWRKY017 | TrWRKY026 | 0.267650903 | 0.835716214 | 0.320265298 |
| TrWRKY017 | TrWRKY053 | 0.841409449 | 4.167421313 | 0.2019017 |
| TrWRKY017 | TrWRKY090 | 0.602483038 | 1.220737087 | 0.493540373 |
| TrWRKY017 | TrWRKY018 | 0.482036879 | 2.592811088 | 0.185912842 |
| TrWRKY017 | TrWRKY030 | 0.604686903 | 1.764411552 | 0.342713072 |
| TrWRKY020 | TrWRKY029 | 0.167810068 | 0.648634964 | 0.258712646 |
| TrWRKY021 | TrWRKY022 | 0.009868563 | 0.048546355 | 0.203281246 |
| TrWRKY023 | TrWRKY029 | 0.02041455 | 0.08739616 | 0.233586345 |
| TrWRKY024 | TrWRKY020 | 0.013850621 | 0.077937963 | 0.177713408 |
| TrWRKY026 | TrWRKY025 | 0.024095337 | 0.085966573 | 0.280287274 |
| TrWRKY028 | TrWRKY139 | 0.780744499 | 3.733449318 | 0.209121494 |
| TrWRKY031 | TrWRKY043 | 0.913135027 | 3.851218311 | 0.237102899 |
| TrWRKY031 | TrWRKY032 | 0.005926932 | 0.044342428 | 0.133662783 |
| TrWRKY031 | TrWRKY091 | 0.614234195 | 1.216020418 | 0.505118323 |
| TrWRKY031 | TrWRKY105 | 0.899393419 | 3.319276274 | 0.270960699 |
| TrWRKY034 | TrWRKY033 | 0.024946231 | 0.06552596 | 0.380707593 |
| TrWRKY035 | TrWRKY097 | 0.718090158 | 2.90894168 | 0.246856155 |
| TrWRKY038 | TrWRKY039 | 0.017248604 | 0.043469432 | 0.396798475 |
| TrWRKY038 | TrWRKY031 | 0.780453386 | 3.31804896 | 0.235214548 |
| TrWRKY038 | TrWRKY036 | 0.555731431 | 1.056182537 | 0.526169872 |
| TrWRKY039 | TrWRKY017 | 0.720899662 | 2.045357895 | 0.352456489 |
| TrWRKY039 | TrWRKY037 | 0.496072102 | 1.479170593 | 0.335371798 |
| TrWRKY043 | TrWRKY042 | 0.004081643 | 0.024795647 | 0.164611263 |
| TrWRKY043 | TrWRKY044 | 0.229740024 | 0.92424277 | 0.248571081 |
| TrWRKY043 | TrWRKY029 | 0.824591772 | 2.332105454 | 0.353582541 |
| TrWRKY045 | TrWRKY044 | 0.009295239 | 0.083601027 | 0.111185704 |
| TrWRKY046 | TrWRKY050 | 0.593700823 | 1.44480974 | 0.410919726 |
| TrWRKY046 | TrWRKY058 | 0.552601042 | 3.050828108 | 0.18113149 |
| TrWRKY046 | TrWRKY054 | 0.574855365 | 2.985913791 | 0.192522425 |
| TrWRKY047 | TrWRKY089 | 0.655874456 | 2.674504017 | 0.245232182 |
| TrWRKY050 | TrWRKY018 | 0.868383493 | 5.634053434 | 0.154131213 |
| TrWRKY054 | TrWRKY055 | 0.009646435 | 0.078032895 | 0.123620112 |
| TrWRKY056 | TrWRKY105 | 0.840349047 | 2.534187833 | 0.331604878 |
| TrWRKY057 | TrWRKY090 | 0.627880141 | 1.645309735 | 0.381618201 |
| TrWRKY057 | TrWRKY063 | 0.009554269 | 0.074014995 | 0.12908559 |
| TrWRKY057 | TrWRKY088 | 0.618094998 | 2.174505321 | 0.284246257 |
| TrWRKY057 | TrWRKY058 | 0.564842582 | 2.140936988 | 0.263829615 |
| TrWRKY057 | TrWRKY029 | 0.743328529 | 2.23560988 | 0.332494742 |
| TrWRKY062 | TrWRKY061 | 0.007723567 | 0.068556488 | 0.112659899 |
| TrWRKY062 | TrWRKY043 | 0.632449198 | 2.43632597 | 0.259591371 |
| TrWRKY064 | TrWRKY063 | 0.491806115 | 1.19181372 | 0.41265351 |
| TrWRKY066 | TrWRKY010 | 0.632885722 | 2.024280848 | 0.312647191 |
| TrWRKY066 | TrWRKY071 | 0.355958512 | 2.980409521 | 0.119432752 |
| TrWRKY066 | TrWRKY045 | 0.671329604 | 3.504621626 | 0.191555516 |
| TrWRKY068 | TrWRKY099 | 0.660905575 | 2.046075749 | 0.323011294 |
| TrWRKY069 | TrWRKY080 | 0.011634905 | 0.124988712 | 0.093087643 |
| TrWRKY070 | TrWRKY139 | 0.684430413 | 5.330569106 | 0.12839725 |
| TrWRKY075 | TrWRKY047 | 0.756430091 | 1.497225778 | 0.505221125 |
| TrWRKY075 | TrWRKY076 | 0.010306489 | 0.025781271 | 0.399766526 |
| TrWRKY079 | TrWRKY086 | 0.504787452 | 2.890380788 | 0.174643927 |
| TrWRKY082 | TrWRKY025 | 0.717727277 | 2.037674862 | 0.352228557 |
| TrWRKY084 | TrWRKY043 | 0.736528909 | 2.027343062 | 0.36329762 |
| TrWRKY084 | TrWRKY029 | 0.760471929 | 4.399425993 | 0.17285708 |
| TrWRKY085 | TrWRKY012 | 0.718425913 | 1.636511369 | 0.438998425 |
| TrWRKY085 | TrWRKY065 | 0.018514697 | 0.058452159 | 0.316749583 |
| TrWRKY085 | TrWRKY087 | 0.464796233 | 1.420098416 | 0.327298607 |
| TrWRKY087 | TrWRKY086 | 0.025969595 | 0.079695758 | 0.325859183 |
| TrWRKY090 | TrWRKY087 | 0.553901085 | 3.175579878 | 0.174425178 |
| TrWRKY091 | TrWRKY022 | 0.736440426 | 1.855584303 | 0.396877913 |
| TrWRKY093 | TrWRKY095 | 0.077992285 | 0.087310984 | 0.893270029 |
| TrWRKY093 | TrWRKY122 | 0.643067602 | 2.475613647 | 0.259760889 |
| TrWRKY093 | TrWRKY063 | 0.797360684 | 2.345245044 | 0.33999035 |
| TrWRKY096 | TrWRKY099 | 0.008457941 | 0.05788793 | 0.146108892 |
| TrWRKY099 | TrWRKY106 | 0.493875965 | 2.363539187 | 0.208956115 |
| TrWRKY099 | TrWRKY044 | 0.734919935 | 2.252137306 | 0.326321106 |
| TrWRKY099 | TrWRKY139 | 0.716342519 | 1.411189821 | 0.507615991 |
| TrWRKY101 | TrWRKY096 | 0.388112143 | 2.056581916 | 0.188717084 |
| TrWRKY104 | TrWRKY105 | 0.010094365 | 0.168589528 | 0.059875398 |
| TrWRKY106 | TrWRKY047 | 0.811075128 | 2.869533967 | 0.282650471 |
| TrWRKY107 | TrWRKY108 | 0.016852275 | 0.084919318 | 0.198450426 |
| TrWRKY109 | TrWRKY060 | 0.746271663 | 2.961610188 | 0.251981732 |
| TrWRKY112 | TrWRKY124 | 0.446362405 | 2.540790868 | 0.17567853 |
| TrWRKY113 | TrWRKY124 | 0.083984235 | 0.146878213 | 0.57179505 |
| TrWRKY113 | TrWRKY118 | 0.566351785 | 1.460399418 | 0.387806088 |
| TrWRKY118 | TrWRKY117 | 0.005339882 | 0.045554797 | 0.117218881 |
| TrWRKY118 | TrWRKY116 | 0.005339882 | 0.045554797 | 0.117218881 |
| TrWRKY123 | TrWRKY105 | 0.678202222 | 1.309962577 | 0.51772641 |
| TrWRKY130 | TrWRKY076 | 0.78506373 | 1.485484435 | 0.528490041 |
| TrWRKY133 | TrWRKY139 | 0.386277766 | 0.826604722 | 0.467306508 |
| TrWRKY134 | TrWRKY125 | 0.46487654 | 1.226735579 | 0.378954151 |
| TrWRKY136 | TrWRKY009 | 0.790729828 | 2.982421145 | 0.265130171 |
| TrWRKY136 | TrWRKY105 | 0.680089483 | 2.654804673 | 0.256173077 |
| TrWRKY139 | TrWRKY132 | 0.728540276 | 2.061932824 | 0.353328813 |
