# Supplementary material for: Genome-wide analysis of the WRKY genes and their important roles during cold stress in white clover
Source: PeerJ. 2023 Jul 11;11:e15610. doi: 10.7717/peerj.15610 (PMC10348312; doi:10.7717/peerj.15610)
Supplement: Supplemental Information 3 [file peerj-11-15610-s003.docx]

**Table S2** AP2 domains present in target genes of *TrWRKY* genes in white clover

| **Gene Locus** | **E-value** | **Score** |
| --- | --- | --- |
| chr1.jg6368 | 2.20E-34 | 111.9 |
| chr13.jg6512 | 5.80E-22 | 72.2 |
| chr11.jg3986 | 1.40E-19 | 64.5 |
| chr3.jg2422 | 2.20E-19 | 63.9 |
| chr15.jg542 | 4.70E-19 | 62.8 |
| chr2.jg2425 | 6.70E-19 | 62.3 |
| chr7.jg5720 | 1.10E-18 | 61.7 |
| chr1.jg8264 | 1.30E-18 | 61.5 |
| chr2.jg6058 | 2.00E-18 | 60.8 |
| chr1.jg9791 | 5.50E-18 | 59.4 |
| chr1.jg1813 | 8.60E-18 | 58.8 |
| chr5.jg426 | 3.00E-16 | 53.9 |
| chr9.jg6482 | 2.50E-15 | 50.9 |
| chr9.jg253 | 6.50E-15 | 49.6 |
| chr5.jg3955 | 6.50E-15 | 49.6 |
| chr5.jg4917 | 1.70E-14 | 48.3 |
| chr11.jg4733 | 1.20E-13 | 45.5 |
| chr5.jg4356 | 1.60E-13 | 45.1 |
| chr5.jg3906 | 3.40E-13 | 44.1 |
