# Supplementary material for: Genome-wide analysis of the WRKY genes and their important roles during cold stress in white clover
Source: PeerJ. 2023 Jul 11;11:e15610. doi: 10.7717/peerj.15610 (PMC10348312; doi:10.7717/peerj.15610)
Supplement: Supplemental Information 4 [file peerj-11-15610-s004.docx]

**Table S3 Raw data of qRT-PCR of *TrWRKY* genes**

| **Name** | **qRT-PCR (Ct value)** | | | |
| --- | --- | --- | --- | --- |
|  | 0h | 0.5h | 1h | 3h |
| *Action* | 33.58 | 28.27 | 31.89 | 35.63 |
|  | 33.38 | 28.83 | 31.68 | 35.63 |
|  | 33.78 | 28.75 | 31.63 | 35.63 |
| *TrWRKY039* | 28.07 | 21.46 | 26.65 | 31.46 |
|  | 27.62 | 20.97 | 25.62 | 30.27 |
|  | 26.59 | 22.09 | 26.98 | 27.94 |
| *TrWRKY041* | 27.83 | 20.56 | 25.90 | 30.47 |
|  | 28.04 | 20.59 | 25.53 | 30.38 |
|  | 28.09 | 20.31 | 25.62 | 30.15 |
| *TrWRKY079* | 33.68 | 23.77 | 30.12 | 34.24 |
|  | 35.18 | 23.66 | 30.19 | 35.16 |
|  | 34.20 | 23.50 | 29.87 | 34.31 |
| *TrWRKY084* | 31.27 | 23.15 | 28.78 | 31.66 |
|  | 31.44 | 23.08 | 28.54 | 32.13 |
|  | 30.80 | 23.04 | 28.34 | 31.51 |
| *TrWRKY100* | 31.81 | 23.43 | 27.04 | 31.21 |
|  | 31.35 | 23.43 | 26.99 | 31.05 |
|  | 30.74 | 23.20 | 26.91 | 30.05 |
| *TrWRKY101* | 30.85 | 23.28 | 26.90 | 30.43 |
|  | 31.09 | 23.33 | 27.03 | 31.21 |
|  | 30.93 | 23.33 | 26.26 | 30.81 |
| *TrWRKY113* | 28.74 | 19.54 | 25.90 | 30.53 |
|  | 28.31 | 19.38 | 25.76 | 29.60 |
|  | 28.13 | 19.17 | 25.86 | 29.58 |
