# Supplementary figures and images for: Genome-wide analysis of the WRKY genes and their important roles during cold stress in white clover
Source: PeerJ. 2023 Jul 11;11:e15610. doi: 10.7717/peerj.15610 (PMC10348312; doi:10.7717/peerj.15610)

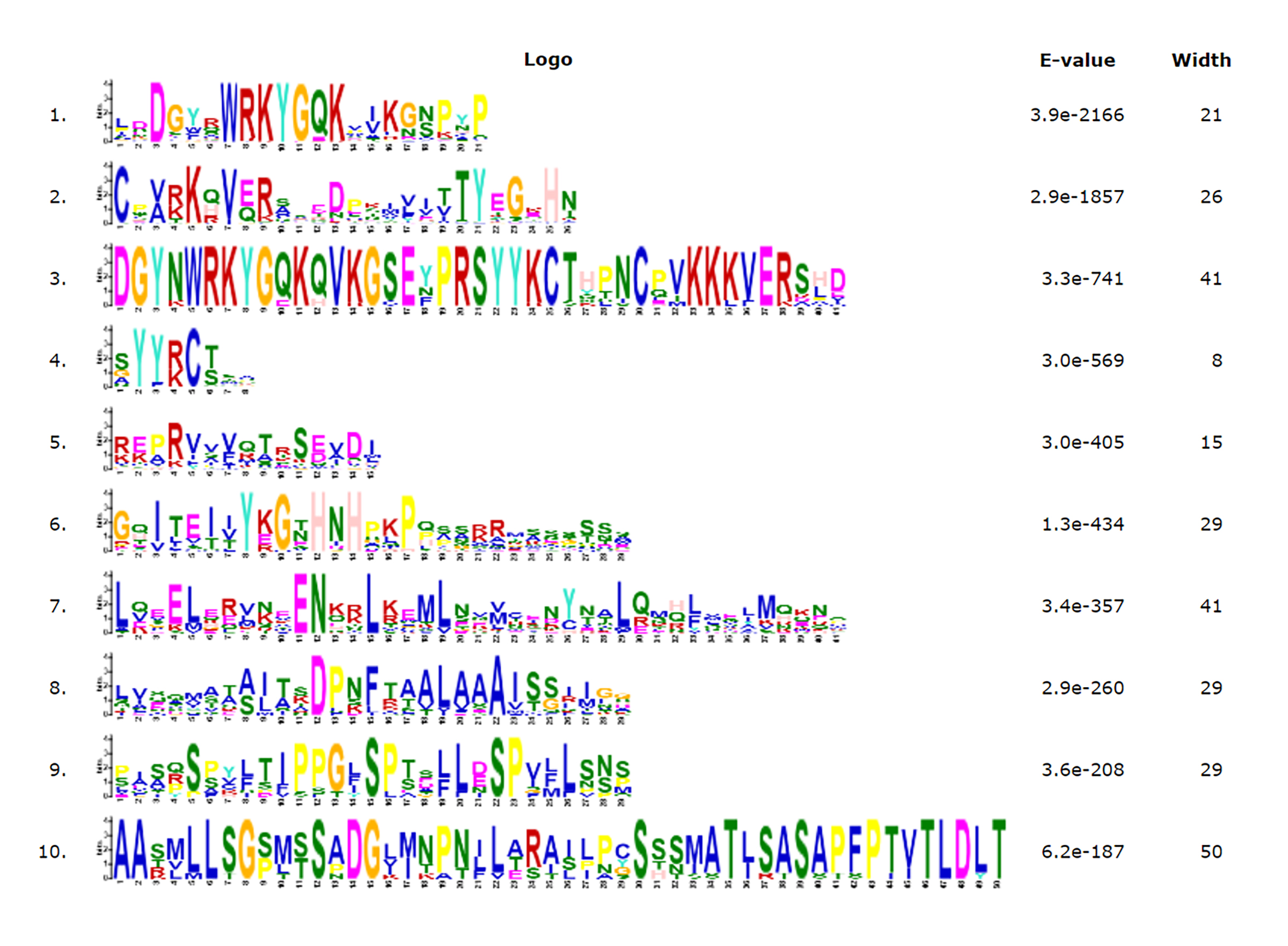

Supplement: Supplemental Information 5 [file peerj-11-15610-s005.jpg]

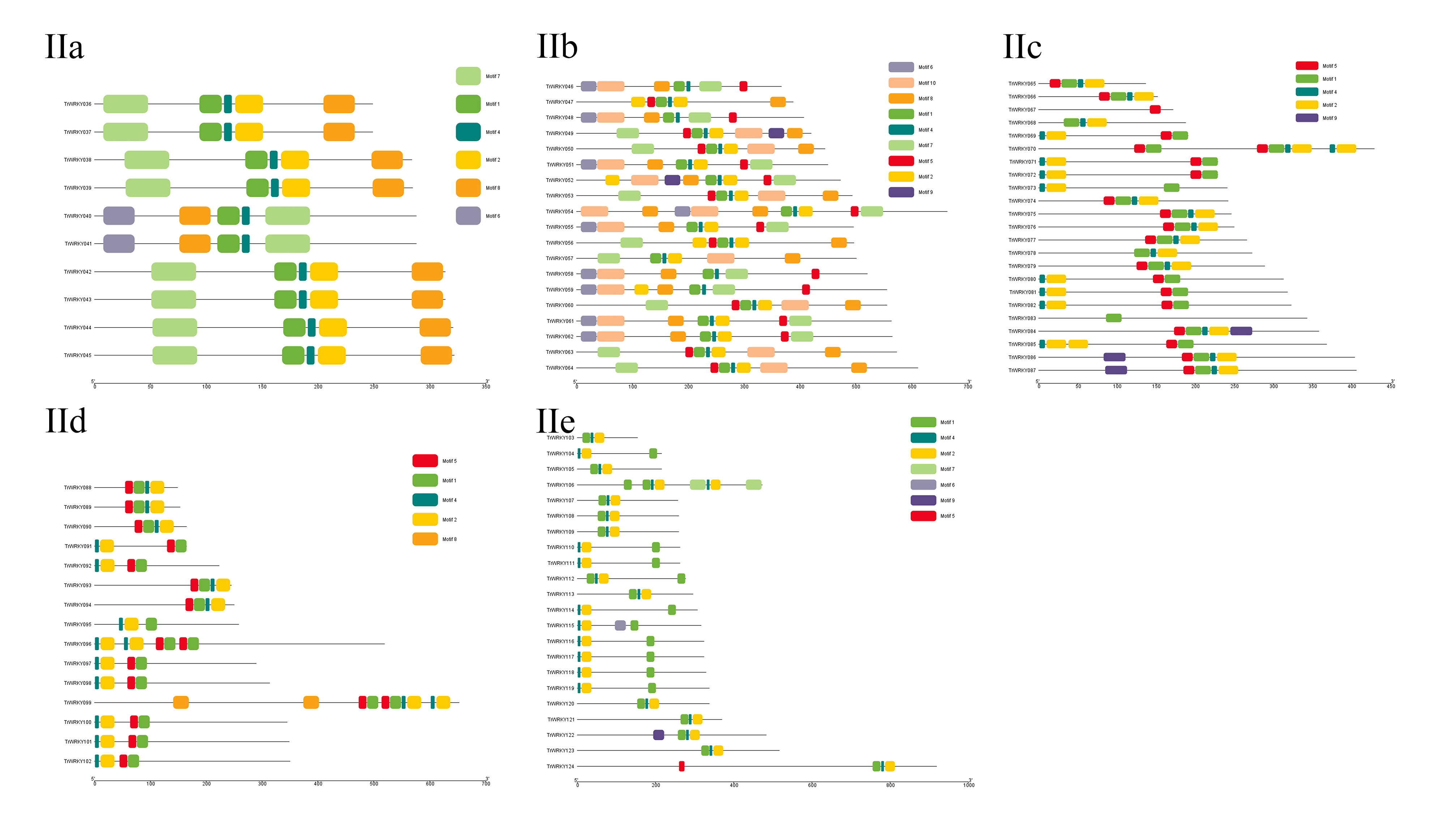

Supplement: Supplemental Information 6 [file peerj-11-15610-s006.jpg]

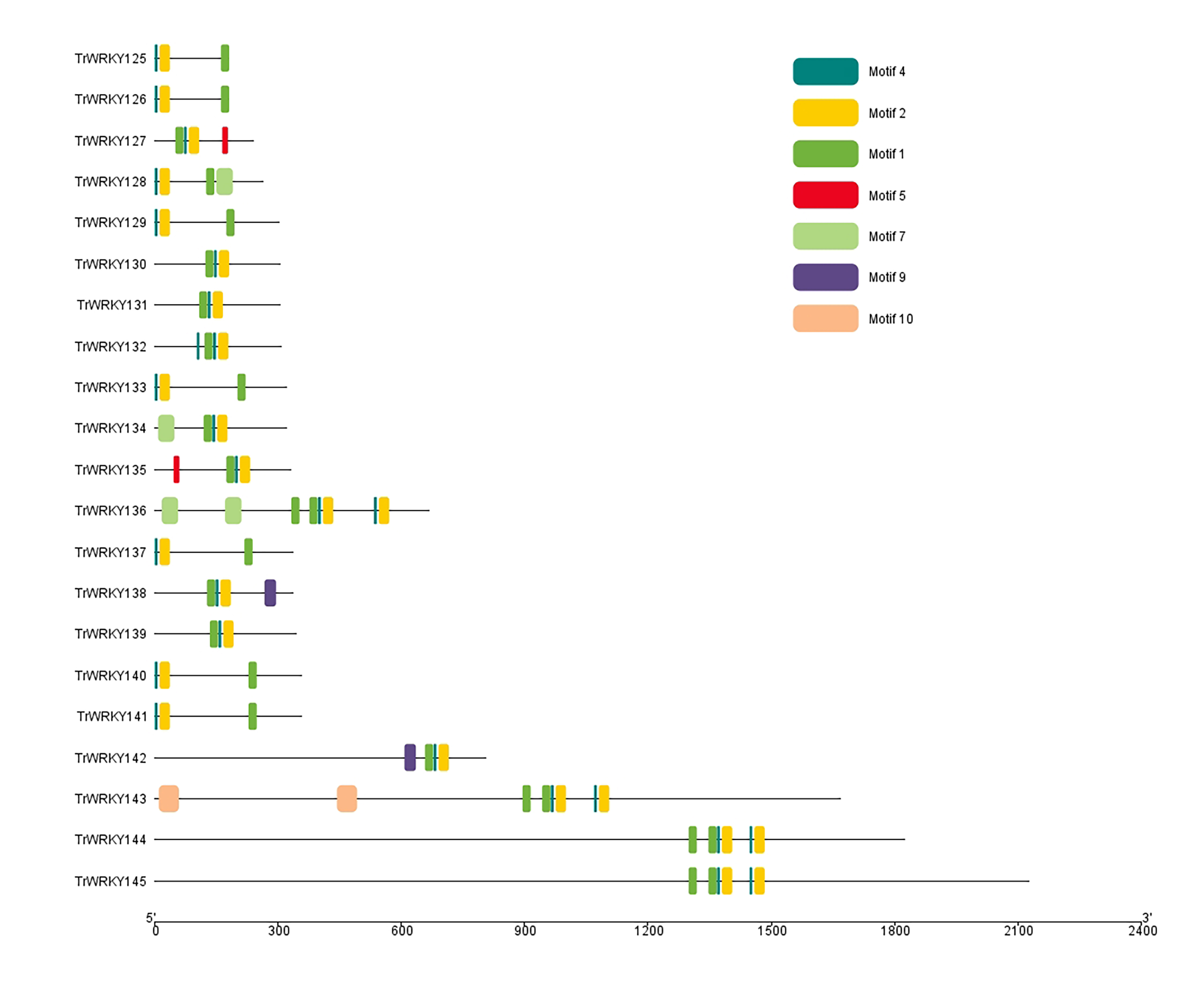

Supplement: Supplemental Information 7 [file peerj-11-15610-s007.jpg]

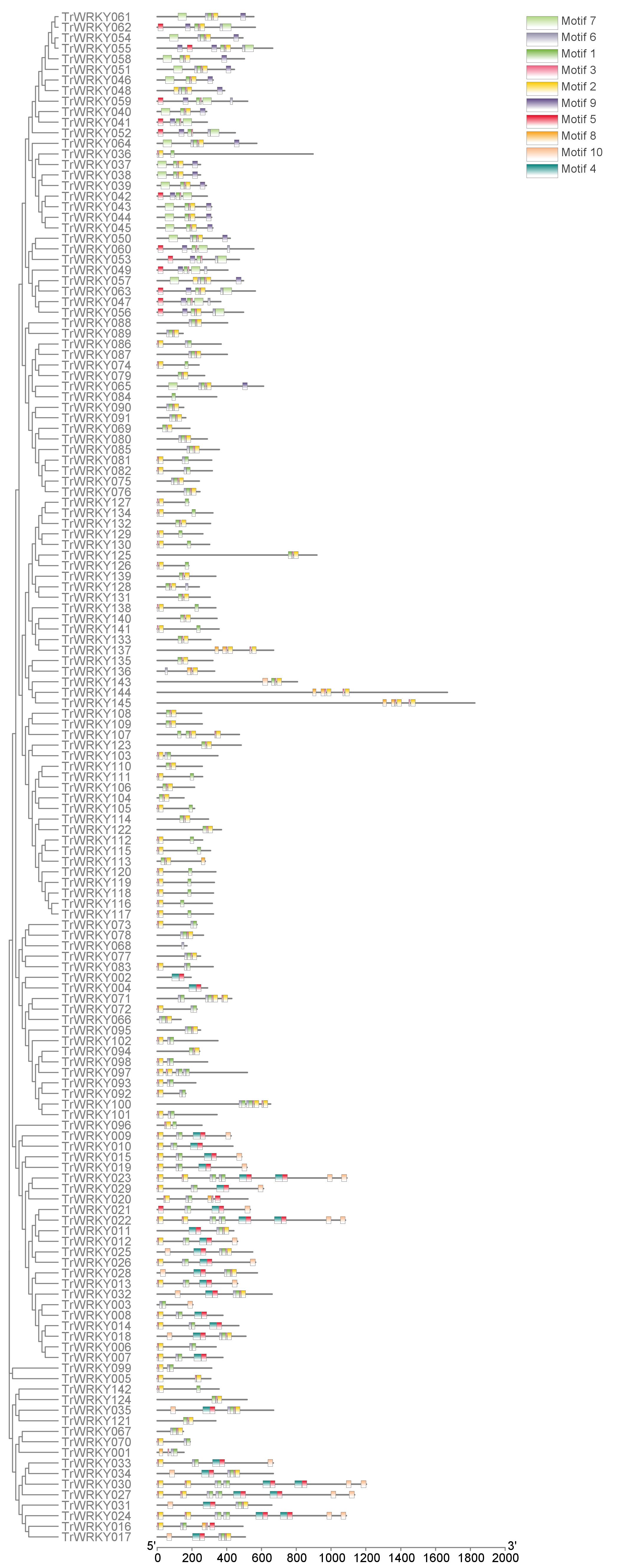

Supplement: Supplemental Information 8 [file peerj-11-15610-s008.jpg]

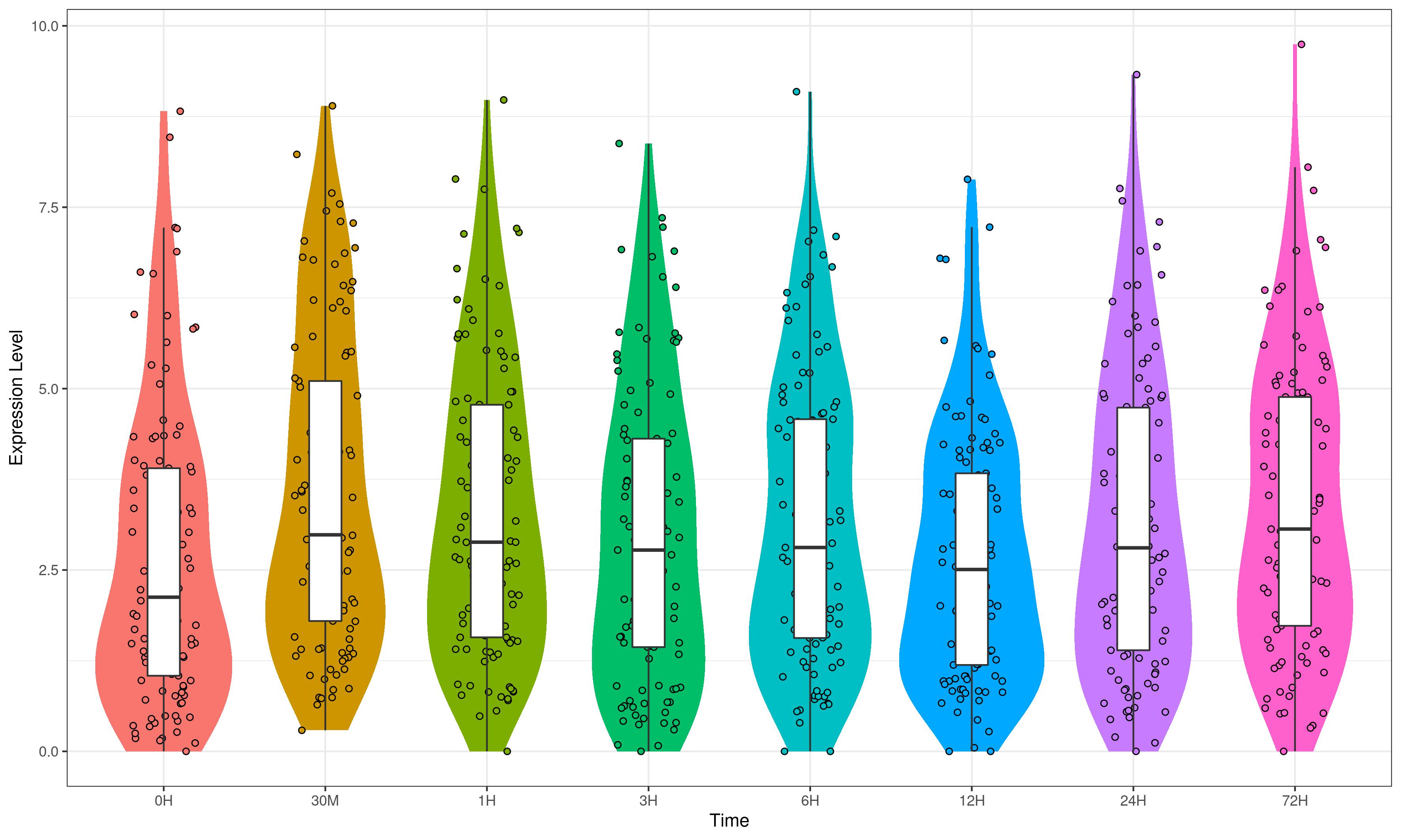

Supplement: Supplemental Information 10 [file peerj-11-15610-s010.jpg]
